# Correlations between the Type of Aggregates in the Bulk Phase and the Functionality and Safety of All-Purpose Cleaners

**DOI:** 10.3390/ijms22126592

**Published:** 2021-06-19

**Authors:** Artur Seweryn, Tomasz Wasilewski, Anita Bocho-Janiszewska

**Affiliations:** Department of Industrial Chemistry, Faculty of Chemical Engineering and Commodity Science, Kazimierz Pulaski University of Technology and Humanities in Radom, Chrobrego 27, 26-600 Radom, Poland; tomasz.wasilewski@uthrad.pl (T.W.); a.janiszewska@uthrad.pl (A.B.-J.)

**Keywords:** all-purpose cleaners, functionality, safety, electrolytes, volume phase, detergency, skin irritation, eco-friendly cleaning products1

## Abstract

The article shows that the type and concentration of inorganic salt can be translated into the structure of the bulk phase and the performance properties of ecological all-purpose cleaners (APC). A base APC formulation was developed. Thereafter, two types of salt (sodium chloride and magnesium chloride) were added at various concentrations to obtain different structures in the bulk phase. The salt addition resulted in the formation of spherical micelles and—upon addition of more electrolyte—of aggregates having a lamellar structure. The formulations had constant viscosities (ab. 500 mPa·s), comparable to those of commercial products. Essential physical-chemical and performance properties of the four formulations varying in salt types and concentrations were evaluated. It was found that the addition of magnesium salt resulted in more favorable characteristics due to the surface activity of the formulations, which translated into adequately high wettability of the investigated hydrophobic surfaces, and their ability to emulsify fat. A decreasing relationship was observed in foaming properties: higher salt concentrations lead to worse foaming properties and foam stability of the solutions. For the magnesium chloride composition, the effect was significantly more pronounced, as compared to the sodium chloride-based formulations. As far as safety of use is concerned, the formulations in which magnesium salt was used caused a much lesser irritation compared with the other investigated formulations. The zein value was observed to decrease with increasing concentrations of the given type of salt in the composition.

## 1. Introduction

All-purpose cleaners (APCs) are products of general use and their main function is to remove all kinds of dirt from various household surfaces. They have to be composed (qualitatively and quantitatively) so as to provide the desirable washing effect on both organically soiled surfaces (for instance, those with remains of food), and environmental dirt (such as dust, soil, rust, or scale). Such products are typically based on a detergent system in the form of an aqueous solution of a mixture of anionic, nonionic, and amphoteric surfactants as well as a variety of additives (preservatives, colorants, fragrance compositions, sequestering agents, and viscosity modifiers) [1,2,3,4].

The main function of the APC products is to provide detergency. Therefore, they are expected to be able to wet the surface they are applied to, penetrate the dirty spot, and disperse it in the washing solution. They must be neutral to all kinds of household surfaces, including laminated, metallic, or wooden ones [1,2,3,4].

Developmental trends in household chemistry indicate a growing interest in formulations based on natural raw materials [5,6,7]. This can be explained in terms of the growing environmental awareness of consumers [8,9,10,11] and various aspects of safety of use [12,13,14,15,16,17,18,19]. As the result, the market is offering an increasing number of products which are entirely based on natural raw materials or have some content of natural additives, such as fragrance compositions [18,19,20]. Moreover, both the consumers and the manufacturers consider it necessary to provide concentrated products. While certain product groups, such as concentrated fiber rinse products [21,22] or hand-dishwashing liquids [15,16,17] do have a concentrated content of active substances, which results in more environmentally friendly formulations, the market of the APC products is known to offer, in addition to genuine concentrates, also pseudo-concentrated products with higher viscosity that makes them look like concentrated products [1,2,3,4,23]. In the commercial practice this can be achieved by using the appropriate viscosity modifier, usually sodium chloride, because it is inexpensive. There have been no reports of any research investigating the effect of such additives on the properties of the washing formulations.

Not many research papers on APCs have been published. Merely a few collective works [1,2,3,4,23,24] and patents [25,26,27,28] on their production technology and composition can be found. No detailed studies on their performance and safety of use, particularly in the context of effect of the type and concentration of their components, have been reported. Most research studies concentrate on testing single compounds or their mixtures [29,30,31,32,33,34,35,36,37]. Only a few reports relate to ready-to-use cosmetic formulations [38,39,40,41] or household chemicals [14] and determine the effect of inorganic viscosity modifiers on product quality.

In this study an attempt was made to determine the impact of inorganic salt type and concentration on the properties of all-purpose cleaners. The APC formulations were developed using vegetable raw materials. Sodium chloride and magnesium chloride were used as the viscosity modifiers. The concentrations of the salts were adjusted to obtain preparations with viscosities comparable to commercial products. The resulting model formulations were analyzed to determine their basic physico-chemical and usable properties such as detergency and foaming ability. Additionally, their safety of use was evaluated.

## 2. Results and Discussion

### 2.1. Development of Formulations and Technologies for the Production of APCs

Based on information from the literature [1,2,3,4,23] and own experience [19,42], the formulations for the starting compositions for making all-purpose cleaning products (detailed recipes and manufacturing methods are presented in the Materials and Methods section) were developed. The inorganic salt type and concentration were analyzed to show impact on the products, specifically on their functional properties and safety of use.

After a literature survey, two inorganic salts—sodium chloride and magnesium chloride which are typical viscosity modifiers in commercial applications—were proposed. Sodium chloride is widely used as a viscosity modifier for washing cosmetics [43,44] and certain household chemicals [12,14,15,16,17,18,37,38,39]. Magnesium chloride is only used as a viscosity modifier in fiber rinse products [21,22]. Moreover, the presence of magnesium chloride in products may increase safety of use in the context of their impact on the skin, according to some literature sources [14,45,46,47].

In the next step of the procedure, a suitable concentration of the viscosity modifiers was selected to obtain the APC formulations. It was assumed that all the formulations would have similar viscosities, of around 500 mPa∙s. Sodium chloride and magnesium chloride were used in this system as viscosity modifiers.

**Figure 1:**
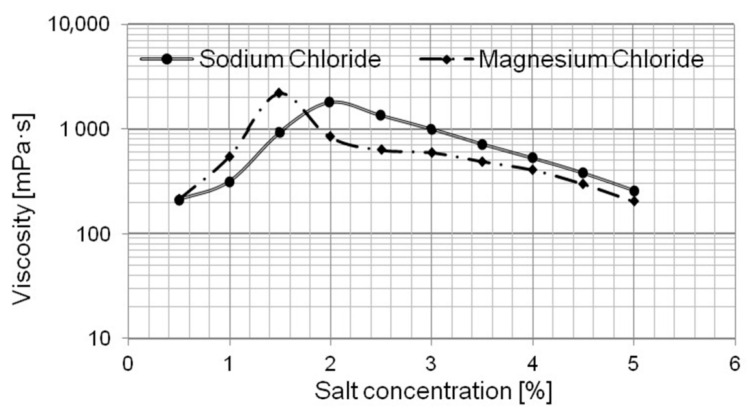
Viscosity of APCs vs. salt concentration for NaCl and MgCl_2_.

In this way two series each of five formulations were prepared, in which the salt concentration was the variable (the tests were performed for the chloride concentration range from 0 to 5% by weight). Introduction of a suitable amount of salt resulted in an adequately lower water content. The appropriate salt was added and mixed until the complete dissolution. In the formulation design process, the so-called *salt curve* was employed [48,49]. The relationship provides information about the impact of the mixture components on viscosity and enables determination of the minimal electrolyte concentration which guarantees the required viscosity. From the technological point of view, the obtaining of a sufficiently high viscosity should not involve too high concentrations of a viscosity modifier, as high electrolyte content in the wash water is known to reduce the critical micelle concentration (CMC), thus leading to an excessive intensification of the product’s detergency. The relationship between viscosity and concentration of the specific chloride was found for the formulations and shown in Figure 1.

Two relationships between dynamic viscosity and salt concentration in the APCs, depending on the salt type (Figure 1) were estimated. The shape of the curves was typical of solutions of surfactants (washing agents). Adding more portions of salt into the solution resulted in the dynamic viscosity increase to a certain maximum of which the value was specific for the given system and after which it decreased. The intensity of viscosity changes depends on the type of salt. For the analyzed compositions, two maximal viscosities were found at a level of ab. 2000 mPa·s (for both salt types). The salt type determines its content necessary for dynamic viscosity to reach the maximum. The thickening effect was more efficient for MgCl_2_—the maximal viscosity of the system was reached at the concentration of salt as low as 1.5%. In the case of sodium chloride, the maximum was obtained at higher concentrations of the modifier, that is, about 2% by weight. The thickening efficiency of sodium chloride to obtain the desirable viscosity is much limited and higher concentrations of the rheology modifier are needed, compared with magnesium chloride.

The base composition for obtaining the APC formulations is a mixture of all types of surfactants (anionic, nonionic, and amphoteric). In the case of the ionic surfactants, micelles having an electrically charged surface are formed in the aqueous solution. On the surface of the charged micelle, there is an electrical double layer, composed of an adsorptive coating (formed by counterions), which is permanently bonded with the micelle, and a diffusion coating which penetrates into the solution. The stability of such micelles depends on the difference in potential between its coatings, the so-called zeta potential. If the zeta potential is different from zero, single-charged aggregates tend to repulse one another electrostatically. By introducing an electrolyte into the micellar solution of the ionic surfactants, the number of counterions both in the adsorptive layer and in the diffusion coating is increased, which reduces the zeta potential. As the micelle charge is changed and the zeta potential is reduced, the electrostatic repulsion forces between the respective micelles tend to disappear gradually. In addition, the repulsive interactions between the hydrophilic *heads* of the surfactant molecules are weakened by the increasing number of counterions bonded to the micelle surface. Consequently, the aggregates become unstable and are transformed into micelles having different structures [49,50,51,52,53,54].

Nonionic surfactants were used in the base composition for the obtaining of APCs because of their high detergency [55,56]. On the other hand, they also have an impact on the micelle shape and size, thus affecting the structure of the bulk phase of the product. Whether the nonionic surfactant micelles formed in the aqueous solution are stable or not depends on the presence of the so-called steric effects between the compound molecules and the solvent. This is connected with hydration of the polyoxyethylene chains and formation of hydrogen bonds between oxygen atoms and water molecules. In this way, a specific spatial barrier which prevents penetration of curved polyoxyethylene chains into one another is created. This barrier provides a stable structure. The introduction of an electrolyte into a nonionic surfactant solution leads to the loss of the steric barrier as the polyoxyethylene chains become dehydrated and smaller. The condition enables the aggregates to approach one another very closely, favoring collisions and joining. Dehydration of a growing number of polyoxyethylene chains makes them occupy a smaller volume in the solution and results in increase of the number of aggregates [49,51,54,55,57,58,59].

The base composition for the obtaining of APCs comprises both ionic and nonionic surfactants. Addition of salt into the system destroys the solvation shell and reduces the zeta potential for the ionic surfactants and dehydrates the polyoxyethylene chains of the nonionic surfactants. Moreover, if a solution is composed of both the nonionic and ionic surfactants, the micelles comprise both groups of the compounds. Incorporation of the nonionic surfactants into the micelle structure of the anionic surfactants results in a reduced zeta potential, thus determining structural changes in the aggregates being formed [49,51,52,56,57,60].

The base composition is characterized by a concentration of the surfactants much above the CMC. In the bulk phase, mainly spherical micelles are formed. They remain in equilibrium with surfactant monomers. The salt added leads to the transformation of the spherical micelles into the cylindrical ones. A solution having this structure is relatively highly viscous. Adding more portions of salt causes further transformation of the micelles into flat structures (so-called *lamellas*) and then multilayered spherical structures, so-called *lamellar droplets*. The transformation is signaled by a reduced viscosity of the system [48,49,56,60,61]. 

An increase in the viscosity of the formulation with increasing concentrations of salt can be explained in terms of the change of the micelle shape from spheres to cylinders, often of much bigger dimensions. The bulk phase may contain even 200 nm long, flexible cylindrical worm-like micelles. The aggregates have a limited ability to move within each other. Moreover, the aggregates are not perfectly straight and may easily be entangled. The presence of these types of structures in aqueous solutions of surfactants results in the systems being highly viscous [62,63,64].

These relationships indicate that the use of a viscosity modifier has a significant impact on the efficiency of structural changes taking place in the bulk phase of the micellar aggregates and also on modifying the viscosity of the system. The ions of multivalent metals, such as magnesium, have a higher binding ability with respect to the charged surface of the micelles of ionic surfactants, thus more efficiently reduce the difference in potential between the Stern layer and the diffusive layer, in comparison with single-valent ions. Consequently, lower concentrations of this type of salt in the solution lead to the decay of electrostatic forces between the hydrophilic *heads* of the surfactants in the micelle and transformation of the aggregates toward the cylindrical structures, which is indicated by increasing viscosities of the system. Moreover, water molecules are strongly bonded by the magnesium ions, which causes dehydration of the polyoxyethylene chains of the nonionic surfactants. This leads to minimization of the steric effects which account for the stability of the micellar aggregates formed, enabling their structural changes in the aqueous solutions. In the case of mixed micelles of both the ionic and nonionic surfactants, the effect which accompanies inhibition of electrostatic interactions between the charged hydrophilic parts of the ionic surfactants as the result of the incorporation of the nonionic surfactants into the micelles, leads to the formation of larger aggregates. The attenuation of electrostatic interactions can be enhanced by introduction of the electrolyte, which creates favorable conditions for the structural changes to take place [29,30,31,32,33,34,35,36,37,39,51,61,65].

In the next step of the study, it was assumed that the thickness of the model products would be comparable to that of commercial products. Based on the obtained relationship (Figure 1), the concentration of the sodium and magnesium salts that guarantees the formulation viscosity comparable to that of commercially available APC formulations was evaluated (on the basis of tests performed, it was found that the viscosity value of commercial products is approx. 500 mPa·s). The required viscosity can be obtained for two concentrations of the salt. In one case, a small amount of the salt added to the composition leads to structural changes in the micelles being formed in the solution, which probably are spherical and have the appropriate size, causing the viscosity of the solution to increase to the desirable value. As more salt is added, the aggregates being formed in the solution tend to change their structure into cylinders—which results in a considerable increase in viscosity—followed by the emergence of lamellar structures as viscosity drops. In this, second, case, the appropriate concentration of salt determines, again, the viscosity of the composition at values which are similar to those recorded for commercially available APCs (Figure 1).

Eventually, a compositional analysis of formulations containing different salt types and concentrations, with viscosities of around 500 mPa·s was carried out. The compositions of the obtained formulations are shown in Table 1.

### 2.2. Appearance and Stability

**Figure 2:**
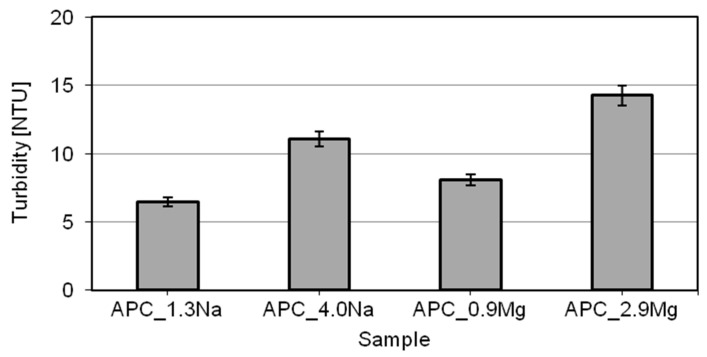
Turbidity of all-purpose cleaners.

The physical-chemical and usable properties of the formulations were tested. The formulations remained microbiologically stable and had the appropriate physico-chemical properties. As viewed by the consumers, the formulations are expected to be clear—this is believed to indicate they are stable. The formulations were tested using a turbidimeter. The results are shown in Figure 2.

**Figure 3:**
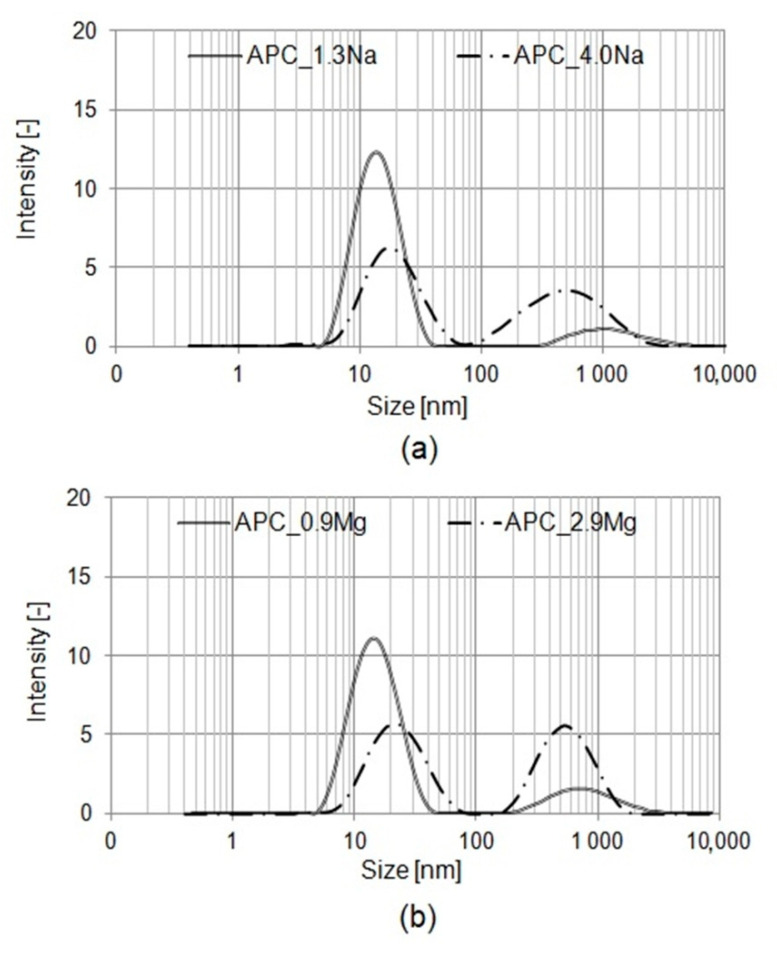
Particle-size distribution of APCs for various salt contents: (**a**) sodium chloride; (**b**) magnesium chloride.

In the turbidity tests, the washing liquids showed values ranging broadly from 6 to 14 NTU. A significant increase in the value of turbidity was recorded for higher concentrations of the given salt type. The turbidity tends to increase gradually with increasing salt concentrations; this was observed for both salts. Differences between the values are attributable to differences between the sizes of the aggregates which are present in the solution. At lower salt contents, the size of the spherical micelles is relatively small, around a few nanometers. This size of micelles does not significantly impact the turbidity of the solution. At higher salt contents, the aggregates formed have the structures of lamellar vesicles or lamellar droplets. Their size is much larger than that of the micelles formed by the surfactants at lower salt contents [29,30,49,66], which results in higher turbidity of the formulations. Moreover, the analyses show that the salt type also has an impact on the turbidity. A comparison of the results of turbidity analyses seems to indicate that the formulations based on magnesium chloride reveal slightly higher turbidities than those based on sodium chloride. The effect is perceptible at both lower and higher salt concentrations. The results correspond to those reported by Ladanyi [67], who observed that at constant concentrations of the surfactants, the aggregates being formed in the presence of bivalent ions originating from the electrolyte were definitely larger than those formed in the presence of sodium cations. The before-mentioned assumptions were verified by a particle-size distribution analysis. Figure 3a,b shows the particle size distributions of compositions containing different salts at various concentrations.

Each of the relationships recorded have two characteristic peaks (Figure 3a,b). In the formulation with 1.3% by weight of NaCl, the first peak, showing the presence of particles of the sizes ranging broadly from 5 to 70 nm seems to indicate the aggregates formed from the molecules of various surfactants used in the formulation. The second peak, having a much lower intensity, relates to particle sizes in the range 200–1200 nm and may indicate the presence in the solution of a small amount of cylindrical micelles and cylinder-like structures of a significant size, termed *worm-like micelles.* The formulation with a much higher content of sodium chloride, showed a lower-intensity peak for particles of sizes between 5 and 70 nm, and the appearance of a high-intensity peak for particles in a wide range between 200 and 1100 nm. The particles of a considerable size which are present in the solution are probably both cylindrical and mono- or multilayered lamellar structures. Similar particle-size ranges were observed in the formulation with magnesium chloride (Figure 3b). However, in comparison with the sodium-chloride based composition, higher intensities were recorded for large particles between a few hundreds and more than a thousand nm. This indicates the formulations have a higher percentage of the larger particles, in comparison with the NaCl-based composition. The effect is observed both in the formulation with a low and a high content of magnesium chloride. The results of the particle-size distribution analysis seem to indicate that the presence in the formulation of magnesium ions determines the formation of the larger aggregates. The statement conforms with data reported in the literature [29,33,49,65,66,67,68,69,70,71].

The results obtained in the analysis correlate with the turbidity test results (Figure 2). The larger particles in the solution are an obstacle to the light wave passage through the system, leading to higher turbidities of the solutions. The higher percentage of large particles, which is observed in formulations with an increased content of (sodium or magnesium) chloride, leads to higher turbidity of the whole system. In the case of magnesium-chloride based APCs, the presence of larger particles in the solution translates into lower clarity, in comparison with the compositions based on sodium chloride.

### 2.3. The Expected Detergency, Based on the Assessment of the Ability to Reduce Surface Tension, Wettability, and Ability to Emulsify Fatty Dirt

The main function of the APCs is the appropriate detergent effect to all kinds of household dirt. Cleaning performance is achieved by selecting the appropriate qualitative and quantitative composition of the formulation. The washing process involves a complex mechanism: it is composed of many phases and is connected with a number of physico-chemical interactions between the surfactants and the dirty surface to be cleaned. Therefore, the composition of the washing formulation must be selected to enable its surfactant component to effectively wet the surface and reduce tension at the dirt-washing bath interface. Consequently, this enables dirt particles to be broken off and removed from the washed surface. The dirt removal from the surface enables the formation of a rather stable emulsion of the hydrophobic dirt and its solubilization by the surfactants which are present in the formulation. The preparation performance depends on a number of partial processes, connected with the washing mechanism (suitable wettability of surface and dirt, ability to emulsify dirt), which are derived from the essential ability of surfactants to lower the interface tension [49,72,73,74,75,76].

**Figure 4:**
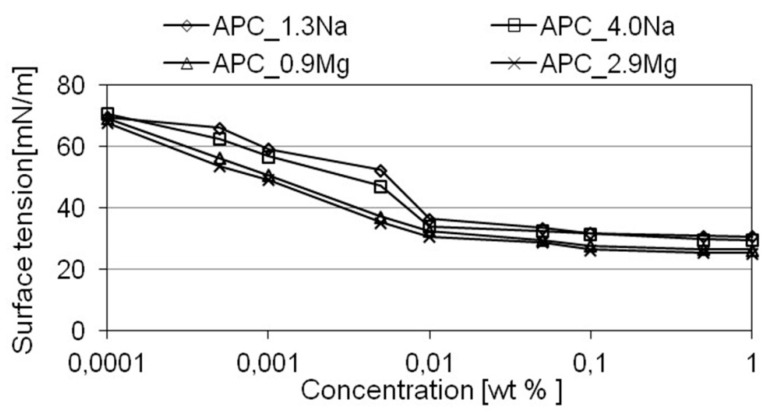
Surface tension vs. concentration of formulation in water.

**Figure 5:**
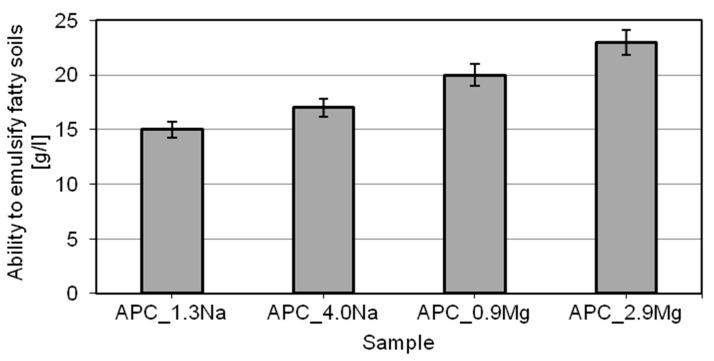
Ability of APCs to emulsify oily dirt.

For the purposes of this study, to evaluate the detergency of formulations containing different types and concentrations of salt, dependence of surface tension at the solution-air interface on concentration of the composition was estimated, the wettability of the washed surfaces was assessed, and the ability to emulsify oily dirt was evaluated. The results are shown in Figure 4 and Figure 5 and Table 2. The relationship between surface tension and concentration of formulations in water is illustrated in Figure 4.

The results obtained fully correspond to those recorded in numerous research works reported in the literature [33,49,51,54,60,61,65,66,67,68,69]. Increasing concentrations of the formulations in water is accompanied by decreasing values of surface tension at the liquid–air interface. The observed relationship has a characteristic profile: as the concentrations of the formulation in water increase, a noticeable, more than two-fold decrease in the value of the parameter in comparison with water was recorded; it was connected with the adsorption of the surfactant monomers at the liquid–air interface. After exceeding a certain concentration, which is typical of the specific system, surface tension takes a comparable value; this indicates that saturation of the adsorption layer has taken place and the micelle formation process has been initiated. This particular concentration is known as the *critical micelle concentration* (CMC). For the systems in question, changes of the parameter vs. concentration of the formulation in the solution varied with the salt type. The solutions with magnesium chloride showed a much higher ability to reduce surface tension, in comparison with the compositions based on sodium chloride. For as low a concentration as 0.05% by weight, the value of surface tension for solutions of the formulation is ab. 25 mN/m. The results are similar to those reported in the literature [33,49,51,54,60,61,65,66,67,68,69], indicating that the presence of bivalent metal ions (such as magnesium or calcium ions) in the solution of ionic surfactants has a significant impact on the association processes taking place in the system and leads to a considerable reduction of the CMC value of the solution. Moreover, according to the literature, if the parameter is reduced, the surfactant solution may have better properties, including detergency, foaming, ability to emulsify, and solubilize hydrophobic substances. Moreover, taking into consideration the concentration of one salt in the formulation, e.g., sodium chloride (APC_1.3 Na and APC_3.9 Na), no significant differences were found between the values of surface tension for solutions of the same salt based formulations at same concentrations in the aqueous solutions.

As far as usable properties are concerned, interesting information was obtained for 1% aqueous solutions of the formulations. At such concentrations, corresponding to the concentration of the commercial APC formulations, the lower values of surface tension were recorded for the magnesium-chloride based solutions which may result in better usable properties of the formulation. The recorded data correspond to the results of the assessment of surface wettability by 1% solutions of the formulations (Table 2).

Table 2 shows wetting angles for 1% solutions of the formulations and various surfaces. The surface types were selected from among materials which are most commonly used in household equipment and which can potentially be cleaned by the APC formulations. Measurements of wetting angles for the 1% aqueous solutions indicate a considerable increase in the affinity of the systems to the surfaces as compared to water. For each of the test solutions, the surface wetting angle was nearly two times as small, in comparison with the value recorded for water, which indicates a much higher affinity between the surfaces and the systems. Differences in the wetting angles of the various surfaces for the same solutions are attributable to their different hydrophobicity and roughness values. The wetting angle of the steel, ceramics and polymer surfaces in contact with water are comparable, whereas that for glass is much lower—both in respect of water and the other test solutions. This might be explained by the high hydrophilicity and low roughness of the glass surfaces.

When comparing the results obtained with the solutions for various types and concentrations of salt, it was observed that wettability of the surfaces in respect of the solutions based on magnesium chloride was higher than that of the solutions based on sodium chloride. The results correspond to those of surface tension for 1% solutions of the formulations. An increased ability of the formulations with magnesium chloride to reduce the value of surface tension translates into better values of the surface wettability of these solutions.

The results are conformable with those reported in a number of research works [77,78,79,80,81,82,83,84], in which the authors indicate a relationship between the surface wettability with the aqueous solutions of the surfactants and different values of surface tensions for these solutions at the liquid–air interface. According to some reports, wettability of hydrophobic surfaces with water can be improved by the effective reduction of the surface tension of water. In their research work on the wettability of various solid surfaces, Zisman et al. [80] introduced the notion of critical surface tension—a parameter which is characteristic of a given surface. They defined it as the surface tension at the liquid–air interface for a given liquid at which wettability of the surface by that liquid is complete. Moreover, they indicate that the surface tension of pure water is much higher than the critical surface tension of most hydrophobic surfaces, therefore, these surfaces are not wetted by water. The authors also maintain that aqueous solutions of surfactants may effectively wet a surface if the compounds in the solution are able to effectively reduce the surface tension of water at the liquid–air interface below the value of critical surface tension which is characteristic of a given surface. Similar conclusions were reported by Dutschk et al. [81,82,83], who studied the wettability of polymer surfaces (polypropylene, teflon, parafilm) by various solutions of ionic surfactants. They demonstrated that the surfactant solutions of which the CMC was much higher than the value of free surface energy of these polymers—and also the value of critical surface tension—are not able to wet this type of surface. On the other hand, nonionic surfactants which are able to effectively reduce the surface tension of water below the value which is typical of a given surface show a certain degree of wettability for this type of substrate. The same tendency was observed by Rafaď et al. [84] in their analysis of wettability of a surface made of ethylene polyterephthalate by aqueous solutions of anionic surfactants. Better wettability with respect to polymer surfaces was observed for solutions with relatively low values of surface tensions.

A very important component of the activities involved in the removal of dirt from various surfaces is the preparation of a sufficiently stable emulsion of the dirt in the washing bath, to enable its effective removal from the washed surface. Whether the process is effective depends largely on the surface properties of the washing bath and that is a consequence of its composition [49,55,72,73,74,75,76]. In this paper, it is indispensable to find out how the ability to emulsify oily dirt is affected by the salt type and concentration. The results of tests of APC formulations demonstrating their ability to emulsify oily dirt are shown in Figure 5.

The APC test formulations have the ability to emulsify oily dirt in the range of 14–23 g/L. Much better oily-dirt emulsification figures were obtained for the compositions based on magnesium chloride, which indicates impact on the test parameter of the chloride type in the formulation. Salt concentration does not appear to have a significant effect on the test results, for either of the two chloride types.

The test results are as expected. As soon as oil is introduced into the system, an emulsion system (a colloid) or, depending on the surfactant type, microemulsion, is formed. Its stability depends on the type and concentration of surfactants and salt in the system [55,85]. According to the literature, after the introduction of bivalent cation ions, emulsion systems show higher stability, which is connected with an effective reduction of CMC of the surfactants and an increased adsorption of the surfactants at the water–oil interface, leading to lower interface tensions and forcing them out toward the water–oil interface. In addition, the bivalent ions tend to hinder the combination of water molecules forming larger aggregates, which limits the contact of water with the dispersed phase [86,87].

### 2.4. Foaming Properties 

**Figure 6:**
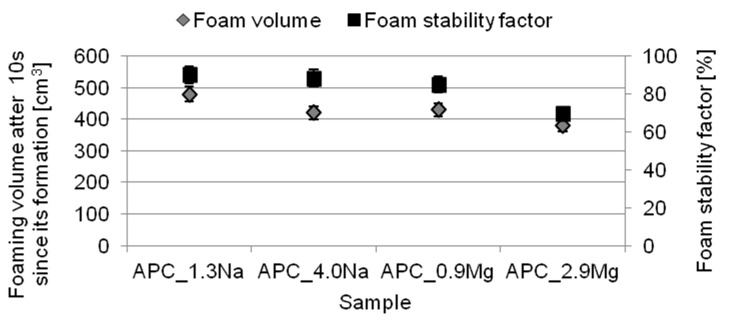
Foam properties of APCs.

The results of examination of foaming properties and foam stability are shown in Figure 6.

The APC formulations have excellent foaming properties. Their aqueous solutions are able to generate a 380–480 cm^3^ volume of foam. The foaming properties of the test formulations were found to depend on the type and concentration of the salt added. Significantly higher values (nearly 25% increase in the foaming properties in comparison with the formulations containing magnesium chloride) were observed for the compositions based on sodium chloride. The results indicate that increasing only the concentration of a given salt type affects the foaming properties of the solutions. 

The foam stability coefficients were also calculated for the formulations. The foam volumes obtained after an interval of time indicate that, even though the formulations with magnesium chloride had better foaming properties, the foam generated in their aqueous solutions was much less stable than that of the compositions with sodium chloride. Their foam stability coefficients were 85% (APC_0.9 Mg) and 70% (APC_2.9 Mg) and approximately 90% for the solutions with magnesium chloride and with sodium chloride, respectively. Moreover, it was observed that increasing the concentration of a given chloride in the formulation resulted in the less stable foams generated; the effect was more perceptible for the solutions with magnesium chloride.

The results obtained are comparable to those reported by Beher et al. [88]. They studied the foaming properties and foam stability of a mixture of Sodium dodecyl benzenesulfonate and Sodium lauryl ether sulfate in the presence of sodium, calcium and aluminum chlorides and found that addition of an electrolyte deteriorated the foaming properties of the systems. The authors pointed out that both the salt type and its concentration had an effect on the phenomenon. Adding a chloride to surfactant solutions improved their surface activity and had a good effect on their adsorption at the liquid–air interface. On the other hand, many reports [65,88,89,90,91,92] indicate that this behavior of surfactants has a positive impact on foaming properties and foam stability in the minimum salt concentration range or just before the CMC of the system. Increased concentrations of salt in the system restrain the saturation of the adsorption layer between the liquid and the gas; this is caused by interactions between the surfactants and the cations originating from the salt and leads to the formation of smaller and less stable air bubbles. Moreover, as suggested by the authors, salt addition has an effect on reduction of the zeta potential in the region, leading to the dispersion being unstable. The efficiency of these processes depends on what type of cations are present in the system and on their affinity for the surfactant molecules. According to these assumptions, foaming properties deteriorate in the series Al^3+^ > Ca^2+^ > Na^+^ in the presence of various salt types. The relationship was confirmed in these studies on APC formulations.

### 2.5. Determination of Irritant Potential—Zein Value (ZV)

The most important aspects of the qualitative evaluation of washing formulations include safety of use, especially in the context of the adverse impact of the products on human skin. Even though persons performing the washing process usually either wear gloves during the activity or use a mop or other specialist equipment for the purpose, studies of consumer preferences relating to household chemicals indicate that only 50% of the consumers protect their hands from contact with the formulations [93]. A considerable percentage of the consumers are exposed to the direct contact with the washing bath. The literature data indicate that many surfactants may interact with the skin, leading to its irritation. The adsorption of monomers from the surfactants onto the skin surface and their interaction with epidermal keratin protein may lead to denaturation of the α-helix structure of keratin. Degradation of the structure of proteins enables them to be washed out of the skin very easily and get solubilized in the solution. In addition, after the evaporation of an excess of water, the swollen proteins of keratin have a lower water bonding ability, resulting in lower degrees of skin wetting and less supple skin. Moreover, the surfactants may interact with the structure of the epidermal intercellular cement, leading to disturbances in its liquid-crystal structure, solubilization of structural lipids and their being washed out of the horny layer, or *stratum corneum.* This may cause delipidation of the horny layer, disturbance of its barrier functions, and higher transepidermal water loss (TEWL) values [11,42,43,94,95,96].

The zein value measurement is a suitable research method enabling determination of the skin irritation potential of surfactants and surfactant-based finished cosmetics and household chemicals according to many reports [11,12,13,14,15,17,18,42,43,94,95]. The method is based on the structural resemblance of the zein protein and the keratin protein building the epidermis. The analysis of interactions between zein and the surfactants is based on determination of the amount of the solubilized, water-insoluble zein protein by the surfactants after its partial denaturation as the result of its contact with the amphiphilic compound in the solution. Zein is hydrophobic and so is keratin in the epidermis. Therefore, interactions between zein and surfactants demonstrate the impact of surfactants on the skin. The essence of the zein value measurement is determination of the amount of solubilized zein in the test solution by measuring, in the test sample, the weight of free nitrogen weight originating from the protein, by the Kiejdahl method [11,42,43,94,95,96,97,98,99,100].

**Figure 7:**
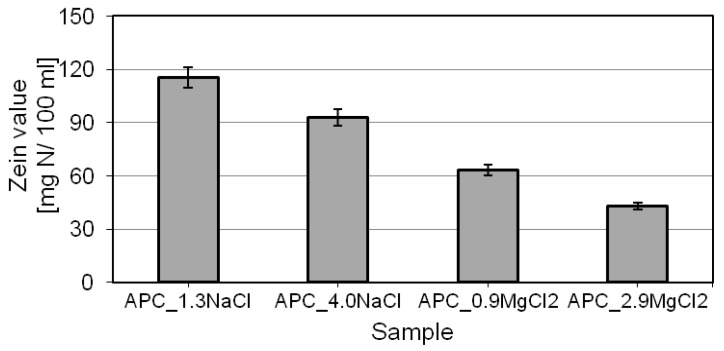
Zein values of APCs.

The results of the zein value determination for the test products are shown in Figure 7.

The model APC test formulations were characterized by rather low zein values (ZV), ranging from 43 to 115 mg N/100 mL. The highest zein value (115 mg N/100 mL) was recorded for the formulation with the lowest content of sodium chloride. For the other formulations, the value decreased. The highest decrease in the zein value, of more than 60% in comparison with the APC_1.3 Na composition, was recorded for the formulation with 2.9% of magnesium chloride. The results indicate that the appropriate salt type and concentration in the formulation are able to significantly minimize their potential irritation to the skin. Introduction of magnesium salt into the system reduces the value of the parameter significantly, indicating an improved safety of products with a content of that salt. Zein has a positive charge at pH 7, therefore, its solubilization efficiency is specifically high for anionic surfactants. These kinds of molecules are able to combine with zein by electrostatic interactions. Zein–surfactant complexes tend to be formed at low concentrations of the anionic surfactants and then, at higher concentrations, a growing number of the attached surfactant molecules initiate zein solubilization within the micellar aggregates of the surfactants. In the case of cationic and amphoteric surfactants, the interactions with zein are much less pronounced because of incompatibility of charges between the surfactant molecules and the protein. Between nonionic surfactants and zein, the electrostatic interactions do not take place [11,43,94,96,97,98,99,100].

Research papers on the subject indicate that zein solubilization in a solution of surfactants is influenced by such factors as chemical structure of the surfactants, intensity of the electrostatic interactions between the zein and amphiphilic molecules. Mixtures of surfactants are observed to have lower CMC values. Moreover, larger and more stable mixed micelles are formed, in comparison with micelles based on a single type of compound, which translates into limited interactions with zein [94,96,97,98,99,100]. An identical effect was achieved with addition of the salts to the APC formulations: higher salt concentrations in the system lead to higher surface activity of the solution and lower CMC (Figure 1). The concentration of free monomers in the solution is minimized, which translates into intensified interactions with the protein. These effects are more explicit for magnesium chloride. Moreover, as found by the particle-size distribution analysis (Figure 3a,b) and from the available data, increased salt concentrations and the salt type determine the formation in the solution of larger and more stable micelles. In the case of magnesium ions, intensified interactions with the surfactants and higher reduction of their electrostatic interactions tend to reduce zein solubilization by the surfactants [11,14,101,102].

## 3. Materials and Methods

### 3.1. Materials

The APC formulations were made using certified, vegetable-based raw materials which are approved for the production of natural products according to COSMOS standards: *Sodium Coco Sulfate* (trade name: Sulfopon 1216 G, supplier: BASF, Ludwigshafen am Rhein, Germany), *Polyglyceryl-4 Laurate/Sebacate (and) Polyglyceryl-6 Caprylate/Caprate (and) Aqua* (trade name: NatraGem S140, supplier: Croda Inc., Snaith, Great Britain), *Coco Glucoside* (Plantacare 818, BASF, Ludwigshafen am Rhein, Germany), *Cocamidopropyl Betaine* (Dehyton K, BASF, Ludwigshafen am Rhein, Germany), *Sodium Benzoate and Potassium Sorbate* as preservative (KEM BS, Akema Fine Chemicals, Coriano, Italy), *Lavender Flower CO_2_-sc extract* as fragrance composition (Flavex Naturextrakte GmbH, Rehlingen, Germany), *Sodium Chloride* (POCH S.A., Gliwice, Poland), *Magnesium Chloride* (POCH S.A., Gliwice, Poland), distilled water. 

The base compositions for the model APC formulations comprised the following: Sodium Coco Sulfate 5%; Coco Glucoside 3%; Cocamidopropyl Betaine 1%; 1% Polyglyceryl-4 Laurate/Sebacate (and) Polyglyceryl-6 Caprylate/Caprate; 1% Sodium Benzoate and Potassium Sorbate, as preservative; 0.25% lavender flower extract (obtained under supercritical CO_2_ conditions) as a fragrance substance with an antimicrobial effect [103,104,105,106,107,108], and water to 100%. 

The formulations were prepared as follows: in the first step, the lavender flower extract was mixed with Polyglyceryl-4 Laurate/Sebacate (and) Polyglyceryl-6 Caprylate/Caprate mixture. In a later phase, this enables the exact solubilization of the hydrophobic extract acting as the fragrance substance with an antimicrobial effect. The two components were mixed at 40 °C. The other components were mixed with water at room temperature. After that, a mixture of a plant-based extract with a solubilizer was added and the resulting system was mixed thoroughly. The solutions obtained in this phase were stable and clear.

### 3.2. Viscosity Measurements

A Brookfield RV DVIII+ rheometer was used. Measurements were carried out at 22 °C with a rotary speed of the cone of 10 rpm. The cone-plate number was CP-52. The viscosities presented in the figures below represent average values obtained from five independent measurements.

### 3.3. Determination of Turbidity

The test was performed using a turbidity analyzer (turbidimeter) HACH 2100 AN. The all-purpose cleaners were transferred to the cuvette which was then placed in the measuring chamber of the turbidimeter. Measurements were carried out at room temperature (~25 °C). The results were evaluated after they had stabilized. The final result was the arithmetic mean of 10 independent measurements [42].

### 3.4. Measurement of Particle-Size Distributions

The particle-size distributions of the APCs were determined by the dynamic light scattering (DLS) technique using a Zetasizer Nano (Malvern, Grovewood Road, United Kingdom) instrument. The size of the particles was measured 24 h after the beginning of production. The measurements were carried out in the range between 1 and 10,000 nm, at a scattering angle of 173° and temperature of 25 °C. The average values from 3 runs with at least 15 measurements each are presented.

### 3.5. Evaluation of the Ability to Emulsify Fatty Soil

The ability to emulsify oily dirt was evaluated in tests conforming to the PN-C-77003 standard. The test consists of determining the maximum weight of rapeseed oil which can be emulsified by 1 L of an APC containing 1 wt. % of the evaluated formulation. The final result (mean value of three independent measurements) obtained in the test was expressed in grams of oil per liter of the evaluated formulation at the concentration of 1 wt. % [42,43]. The test methodology was described by Seweryn et al. [14] and Wasilewski et al. [15,17,18].

### 3.6. Surface Tension Measurements

The surface tension of aqueous solutions of the formulations was measured by the ring tear-off method using the TD 1C tensiometer from Lauda (measurement range: <300 mN/m), equipped with a platinum Du Noüy ring according to DIN 53914 and ASTM D971 (ring radius R = 9.55 mm, ring wire radius r = 0.20 mm). Aqueous solutions of the formulations were analyzed at the following concentrations: 0.001, 0.005, 0.010, 0.055, 0.100, 0.500, and 1.000%. Measurements were carried out at room temperature (~25 °C). The results, shown in the diagram, are arithmetic means from 10 independent measurements per sample.

### 3.7. Contact Angle

The wettability of various materials was determined using a drop shape analysis technique. A KRÜSS DSA100 instrument was used for the tests, which were carried out at 25 °C. The wetting angles of metal (steel 100Cr6, surface roughness R_a_ = 0.043 μm), ceramic (Al_2_O_3_), glass (silicate glass), and polymer (PMMA-Poly(methyl methacrylate)) surfaces were measured. 

### 3.8. Evaluation of Foaming Properties

The method of measurement was in line with the European Standard CSN–EN 12728. The experiments were carried out as follows: 100 cm^3^ of 1% aqueous solution of the investigated all-purpose cleaner was poured into a glass cylinder. Then, it was whipped to produce foam (time of whipping 60 s, number of full hits 60) using a perforated disc fixed to a metal bar. The volume of the foam formed was read out after 10 s. Foaming ability is described as the foam volume after 10 s since its formation. Additionally, the percent foam stability coefficient was evaluated as the ratio of the foam volume after 10 min and after 1 min. Measurements were carried out at room temperature (~25 °C). The final result was the arithmetic mean of three independent measurements [42,43]. The test methodology was described by Seweryn et al. [14] and Wasilewski et al. [15,17,18].

### 3.9. Determination of Irritant Potential—Zein Value (ZV)

In the zein test procedure, 2 g of protein is solubilized in 40 g of a test sample of the solution of the all-purpose cleaner formulation (10% wt.). The amount of the solubilized protein was determined by Kjeldahl analysis, and the result of the zein value procedure was expressed as mg of the solubilized protein in 100 mL of the sample. The final result was the arithmetic mean of three independent measurements [42,43]. The test methodology was described by Seweryn et al. [14] and Wasilewski et al. [15,18].

### 3.10. Error Analysis

The points in the charts represent mean values from a series of three or five independent measurements. The t-distribution was used to calculate confidence limits for the mean values. Confidence intervals, which constitute a measuring error, were determined for the confidence level of 0.90. Error values are presented in the Figure 2, Figure 4, Figure 5 and Figure 6.

## 4. Conclusions

The effect of the type and concentration of inorganic salt on the properties of all-purpose cleaners was analyzed in this paper. In the first step, the authors determined the concentration of the specific type of salt which was necessary for obtaining a composition of which the viscosity was similar to that of formulations available on the retail market. It was found that the salt concentration which is required for obtaining formulations with the specific viscosity, was determined by the salt type. In the case of magnesium salt, the required viscosities were obtained at much lower salt concentrations, indicating that the system was more responsive to modification of its viscosity with magnesium chloride. Physico-chemical analyses and performance tests were carried out for the prepared model products, and the results indicate that:

- electrolyte type has an effect on the surface activity of formulations in aqueous solutions. Addition of magnesium chloride into the compositions reduced the surface tension of their aqueous solutions. The effect of the specific salt type concentration on the parameter in question was not determined;
- particle-size distribution analyses indicate that the salt type and concentration had an effect on the size and type of aggregates being formed in the solution. The systems with magnesium chloride were shown to form large aggregates in comparison with those containing sodium chloride. The results correspond to those obtained in the turbidity tests;
- the formulations are characterized by sufficiently high wettability with respect to hydrophobic surfaces. Measurements of the wetting angle indicate that the composition with magnesium chloride has higher wettability in comparison with the formulations with sodium chloride. The effect of concentration of the specific chloride on the investigated parameter was not determined. The results correspond to those obtained in surface tension measurements for aqueous solutions of the formulations;
- emulsification properties of the model products increase with increasing concentrations of the chloride type used. Moreover, much better characteristics relating to the ability to emulsify hydrophobic dirt was recorded in the presence of magnesium chloride;
- the formulations revealed good foaming properties. Moreover, for increasing concentrations of the specific chloride type, the solutions were characterized by worse foaming properties and foam stability. The compositions with magnesium chloride showed worse foaming properties in comparison with the systems containing sodium chloride;
- the type and concentration of the electrolyte used determine the zein value. For increasing concentrations of the given salt, the parameter was observed to decrease. In the presence of double-charged cations (Mg^2+^), the test compositions proved to be definitely better in the aspect of irritation potential. For such formulations, the zein value was nearly 60% lower than that of the compositions containing sodium chloride.

## Figures and Tables

**Table 1:** Model formulations of APCs.

Name according to INCI ^1^	Concentration [wt. %]			
	APC_1.3 Na	APC_4.0 Na	APC_0.9 Mg	APC_2.9 Mg
Sodium Coco Sulfate	5.00	5.00	5.00	5.00
Coco Glucoside	3.00	3.00	3.00	3.00
Cocamidopropyl Betaine	1.00	1.00	1.00	1.00
Polyglyceryl-4 Laurate/Sebacate (and) Polyglyceryl-6 Caprylate/Caprate	1.00	1.00	1.00	1.00
Sodium Chloride	1.30	4.00	-	-
Magnesium Chloride	-	-	0.90	2.90
Citric Acid	0.50	0.50	0.50	0.50
Lavender Flower CO_2_ Extract	0.25	0.25	0.25	0.25
Sodium Benzoate (and) Potassium Sorbate	0.10	0.10	0.10	0.10
Aqua	to 100	to 100	to 100	to 100

^1^ INCI = International Nomenclature of Cosmetic Ingredients.

**Table 2:** Wetting angle of APCs as 1% solutions for various surface types.

Surface	Wetting Angle Ө [deg]				
	Water	APC_1.3 Na	APC_4.0 Na	APC_0.9 Mg	APC_2.9 Mg
Steel	82.1 ± 0.23	49.7 ± 0.69	41.08 ± 0.54	40.9 ± 0.66	40.8 ± 0.66
Ceramics	75.4 ± 0.87	51.0 ± 0.26	44.3 ± 0.19	40.9 ± 0.74	36.8 ± 0.88
Glass	21.5 ± 0.55	16.5 ± 0.68	13.2 ± 0.40	13.4 ± 0.91	12.7 ± 0.64
Polymer	78.5 ± 0.12	50.9 ± 0.31	48.2 ± 0.56	44.7 ± 0.12	43.6 ± 0.62

## Data Availability

Data are contained within the manuscript.
